# The production of concentrated dispersions of few-layer graphene by the direct exfoliation of graphite in organosilanes

**DOI:** 10.1186/1556-276X-7-674

**Published:** 2012-12-13

**Authors:** Daniele Nuvoli, Valeria Alzari, Roberta Sanna, Sergio Scognamillo, Massimo Piccinini, Laura Peponi, Josè Maria Kenny, Alberto Mariani

**Affiliations:** 1 Dipartimento di Chimica e Farmacia, Università di Sassari and local INSTM Unit, Via Vienna 2, Sassari, 07100, Italy; 2 Porto Conte Ricerche S.r.l., SP 55 km 8.400 Loc., Tramariglio, Alghero, Sassari, 07041, Italy; 3 ICTP-CSIC, Juan de la Cierva 3, Madrid, 28006, Spain

**Keywords:** Graphene, Organosilanes, High concentration, Liquid exfoliation

## Abstract

We report the formation and characterization of graphene dispersions in two organosilanes, 3-glycidoxypropyl trimethoxysilane (GPTMS) and phenyl triethoxysilane (PhTES) as new reactive solvents. The preparation method was mild and easy and does not produce any chemical modification. The dispersions, which exhibit the Tyndall effect, were characterized by TEM and Raman spectroscopy to confirm the presence of few-layer graphene. Concentrations as high as 0.66 and 8.00 mg/ml were found for PhTES and GPTMS, respectively. The latter is one of the highest values reported for a dispersion of graphene obtained by any method. This finding paves the way for the direct synthesis of polymer nanofiller-containing composites consisting of graphene and reactive silanes to be used in sol–gel synthesis, without any need for solvent removal, thus preventing graphene reaggregation to form graphite flakes.

## Background

Graphene, with its unique physical properties [1-4], is one of the most exciting nanomaterials discovered in the last years. A particularly promising graphene production technique is based on the obtainment of colloidal suspensions from graphite, or its derivatives [5]. In spite of other methods like epitaxial growth [6], chemical vapor deposition [7], and micromechanical exfoliation [8], this approach is both scalable, affording the possibility of high-volume production, and versatile in terms of chemical functionalization, which, on the other hand, is sometimes exploited to favor graphene obtainment and its dispersion. Colloidal graphene suspensions may be advantageous in that they could be used for a wide range of applications including: spray coating, vacuum filtration, or drop casting; moreover, by mixing them with polymers, graphene-based polymer composites can be prepared. In these production methods, the key challenge involves the exfoliation of graphitic materials in a liquid that can disperse graphene sheets in a stable way.

One of the most common methods to obtain graphene by this technique involves the dispersion and exfoliation of graphene oxide [5]. This material consists of graphene sheets which are chemically functionalized with groups such as hydroxy and epoxide, which stabilize them in water. However, this functionalization results in considerable damaging of graphene electronic structure, which becomes a semiconductor. While the epoxide functionalities can be removed by reduction [9], the hydroxy and carboxy cannot be removed [10]. Recently, a significant breakthrough was made when some research groups showed that graphite could be exfoliated by non-chemical solution phase methods with solvents [11-13] or surfactants [14,15]. The energy required to exfoliate graphene is balanced by the solvent-graphene interaction for compounds whose surface energy matches that of graphene [11]. By this technique, our research group obtained few-layer graphene sheets by sonication of graphite with *N*-methyl pyrrolidone [16], isocyanates [17], *N*-vinyl caprolactam [18], a commercial ionic liquid [19], and an acrylate [20], thus, achieving a graphene concentration as high as 2.21, 3.78, 5.00, 5.33 and 9.45 mg/ml, respectively, which are among the highest reported so far in any liquid.

Analogously in the present work, we used reactive organosilane compounds to obtain stable graphene dispersions. Organosilanes are characterized by the basic structure R_*n*_Si(OR)_4 − *n*_, where R is an alkyl, aryl, or organofunctional group, and OR is generally a methoxy or ethoxy group, which can react with the hydroxyl groups and liberate methanol or ethanol. Organo(alkoxy)silanes are used in coating applications for surface treatment, as additives in paints, inks, and adhesives, and as reactive intermediates for silicone resin syntheses and organic resin modifications: The alkoxy group can be exploited for the linkage with inorganic substrates, pigments, fillers and hydroxy functionalized polymers. Organo(alkoxy)silanes are used as precursors for the synthesis of inorganic–organic hybrid materials [21]. In particular, 3-glycidoxypropyl trimethoxysilane (GPTMS) and phenyl triethoxysilane (PhTES) are two of the most commonly used for these purposes. Hydrolysis of the methoxy or ethoxy groups of GPTMS and PhTES gives rise to silanol groups which can condense to form silicate networks [22]. Moreover, GPTMS has an epoxy ring that can be used for organic network formation, for instance, through condensation with diethylenetriamine or ethylenediamine at room temperature; this dual functionality makes GPTMS a strategic component for the preparation of polymer hybrids [23].

In the present work, for the first time PhTES and GPTMS were used for dispersing graphene by direct graphite sonication, without any chemical manipulation. The resulting material was thoroughly characterized by using Raman and UV spectroscopies. Moreover, transmission electron microscopy was used for the statistical analysis concerning the distribution among mono and few-layer graphenes.

## Methods

### Materials

Phenyl triethoxysilane, 3-glycidoxypropyl trimethoxysilane and graphite flakes (+100 mesh) were purchased from Sigma Aldrich (Sigma-Aldrich Corporation, St. Louis, MO, USA) and used as received without further purification.

### Graphene dispersions

Mixtures containing various amounts of graphite flakes and organosilane (5.00 g) were put in a tubular plastic reactor (*i.d.* 15 mm) and placed in an ultrasonic bath (0.55 KW, water temperature ≈ 25°C) for 24 h. Then, after they were centrifuged for 30 min at 4,000 rpm, the gray to black liquid phase containing graphene was recovered.

### Graphene dispersion concentration

In order to determine the graphene concentration, the above dispersion was divided into two fractions with a known volume. The first one was filtered through polyvinylidene fluoride filters (PVDF, pore size of 0.22 μm) in order to directly weigh the amount of dispersed graphene and determine the actual graphene concentration.

### Determination of absorption coefficient *α*

The second aliquot of the above dispersion was analyzed by UV analysis with a Hitachi U-2010 spectrometer (1 cm cuvette, Hitachi Corporation, Tokyo, Japan). The above gravimetric data allowed us to determine the absorption coefficient *α*: from a known volume of initial dispersion, several dilutions were done and the absorbance at a wavelength of 660 nm was measured [11,17,24]. Absorbance versus concentration plots gave the absorption coefficient *α* (see Results and discussion) value.

### TEM analyses

TEM measurements were performed on a JEOL JEM-2100 TEM instrument (JEOL Ltd., Akishima, Tokyo, Japan), with a LaB6 filament, with an operating voltage of 200 kV.

For the TEM analysis, the solutions were sonicated for 5 min and then cast directly on the 200 mesh cooper grid; eventually, the solvent was evaporated at ambient conditions for 24 h.

### Raman spectroscopy

Analyses were performed on graphene flakes obtained after vacuum filtration of dispersions on PVDF filters (pore size 0.22 μm), with a Bruker Senterra Raman microscope (Bruker Corporation, Billerica, MA, USA), using an excitation wavelength of 532 nm at 5 mW. The spectra were acquired by averaging five acquisitions of 5 s with a ×50 objective.

## Results and discussion

The feasibility of using organosilanes as effective liquid dispersing media for graphene was investigated. In particular, PhTES and GPTMS were tested with the aim of preparing graphene dispersions that could be directly utilized for further uses without recovering graphene in the solid state, thus avoiding any possible restacking of it to pristine graphite and compromising any previous successful exfoliation process.

**Figure 1:**
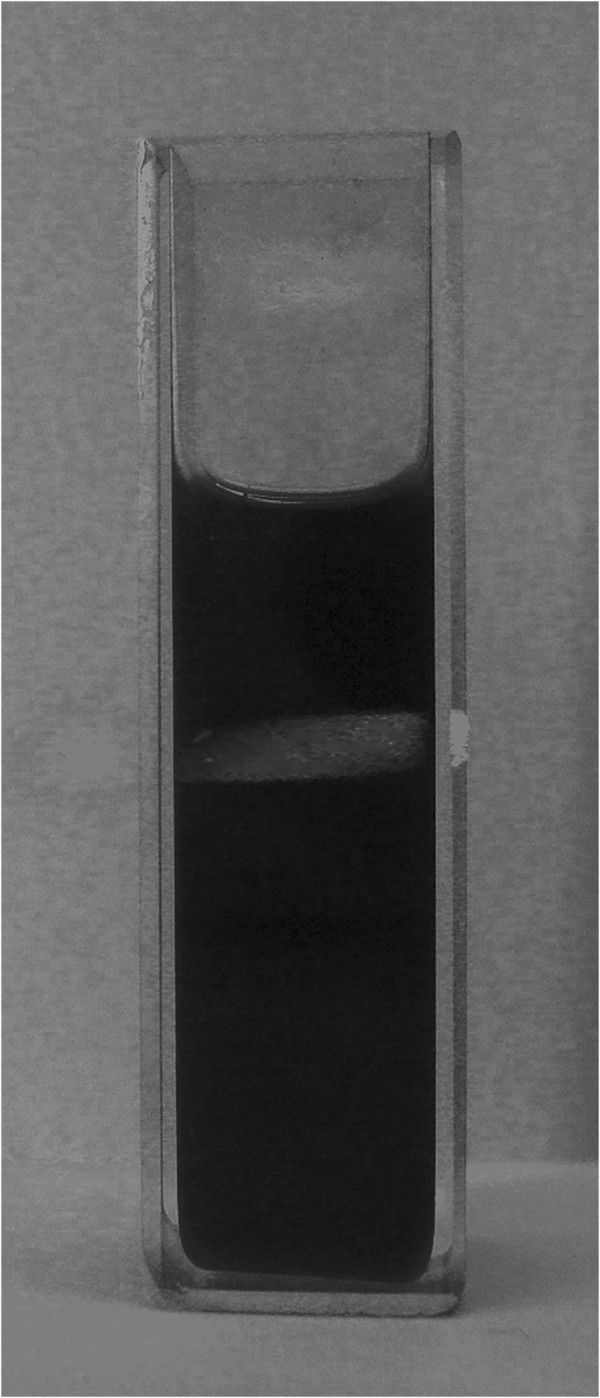
**Tyndall effect exhibited by a graphene dispersion in GPTMS.** When the red laser light passes through the dispersion, it is scattered and becomes visible.

As described in ‘Methods’ section, the procedure used was as simple as possible and envisaged the direct sonication of graphite without any chemical manipulation. A first indication of the nanometric dimensions of the dispersed graphene particles was provided by the occurrence of the Tyndall effect [25]. Both GPTMS (Figure 1) and PhTES dispersions exhibit graphene light scattering, thus confirming the colloidal nature of these systems.

**Figure 2:**
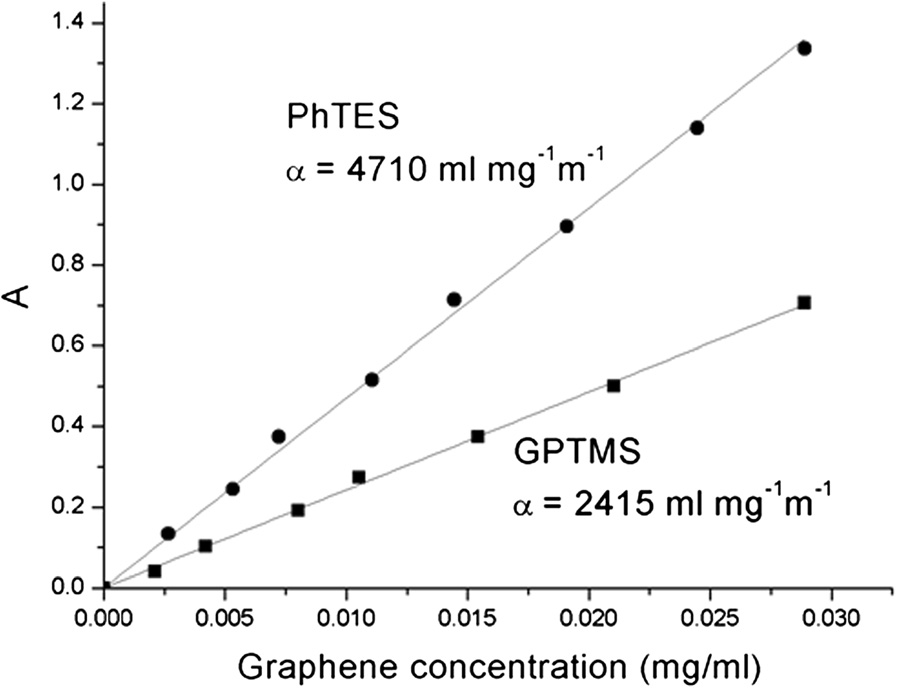
**Absorption coefficient *α *determination.** Optical absorbance (660 nm) as a function of graphene concentration in PhTES and GPTMS. A Lambert-Beer behavior is shown, with an absorption coefficient *α* of 4,710 and 2,415 ml·mg^−1^·m^−1^, respectively.

With the aim of determine the best initial graphite/liquid medium ratio that allows obtaining the highest graphene concentration, a series of dispersions with different amounts of graphite was prepared for each of the two organosilanes. In order to find the absorption coefficient *α* and set-up a reliable method for the determination of graphene concentration in the above media, UV and gravimetric analysis were carried out (see ‘Methods’ section). Figure 2 shows the Lambert-Beer behavior and the different slopes of the two suspensions, thus indicating that the two media have different dispersibility. Namely, for GPTMS and PhTES, an absorption coefficient of 2,415 and 4,710 ml·mg^−1^·m^−1^ was respectively found.

**Figure 3:**
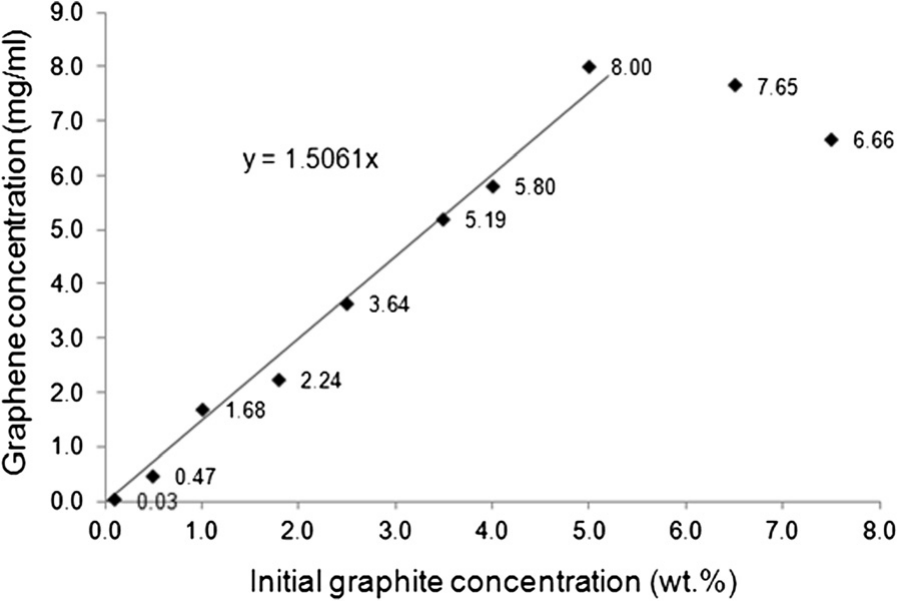
**Graphene concentration in GPTMS as a function of initial graphite concentration.** Sonication time = 24 h.

**Figure 4:**
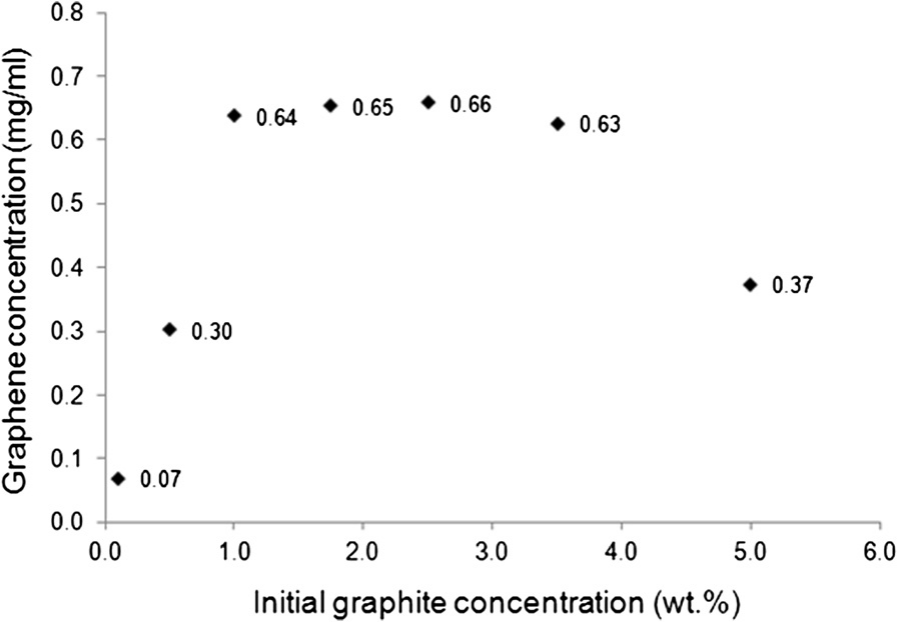
**Graphene concentration in PhTES as a function of initial graphite concentration.** Sonication time = 24 h.

The concentrations of graphene in GPTMS and PhTES as functions of the initial graphite concentration are reported in Figures 3 and 4, respectively. As far as the GPTMS dispersion is concerned, a direct proportionality between initial graphite and graphene seems to exist up to 5 wt.% of the initial graphite (Figure 3). After this value, a decrease of graphene concentration was found. This is probably due to the following observed phenomenon: When high concentrations of graphite are added to the used plastic reactor, it tends to precipitate thus making the subsequent sonication process less effective. It should be highlighted that the maximum concentration of graphene here obtained is one of the highest reported so far by any method [11,21,26,27]. An analogous trend was observed also in the case of PhTES (Figure 4), for which the maximum graphene concentration (0.66 mg/ml) was found when the initial graphite concentration was 2.5 wt.%. Besides, the maximum amount of graphene that can be dispersed in PhTES is much lower than in the case of GPTMS, 0.66 mg/ml instead of 8.0 mg/ml, thus indicating that this latter liquid medium is more effective than PhTES in the dispersing graphene.

**Figure 5:**
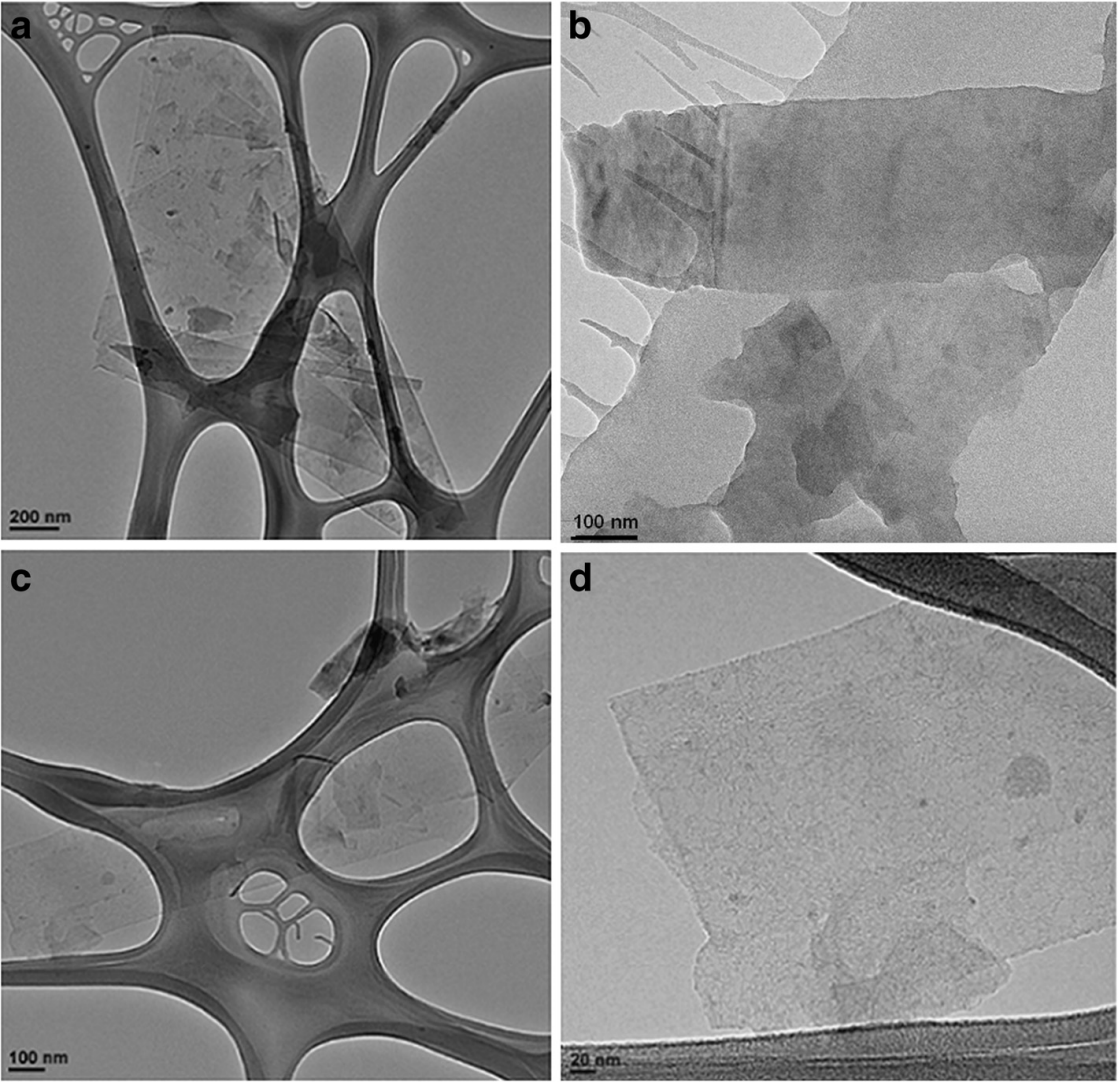
**TEM micrographies.** Graphene dispersed in GPTMS (**a**,**b**) and in PhTES (**c**,**d**).

TEM was used to investigate the state of the graphene particles dispersed in organosilanes; indeed, this technique is usually employed for the investigation of graphene dispersions [11,12,14,23]. As shown in Figure 5, the images revealed a large quantity of flakes of different types. A larger proportion of flakes were few-layer graphene of various dimensions: in particular, very large flakes (lateral size approximately 1 μm) and smaller flakes with an average lateral size of 100 to 200 nm. It should be underlined that, in all cases, we did not observe graphite aggregates. Despite of what was reported in other applications, a relatively large size distribution is generally not considered a drawback in polymer nanocomposites in which the nanometric dimensions of the filler is the predominant factor influencing the properties of the resulting material [16,18].

**Figure 6:**
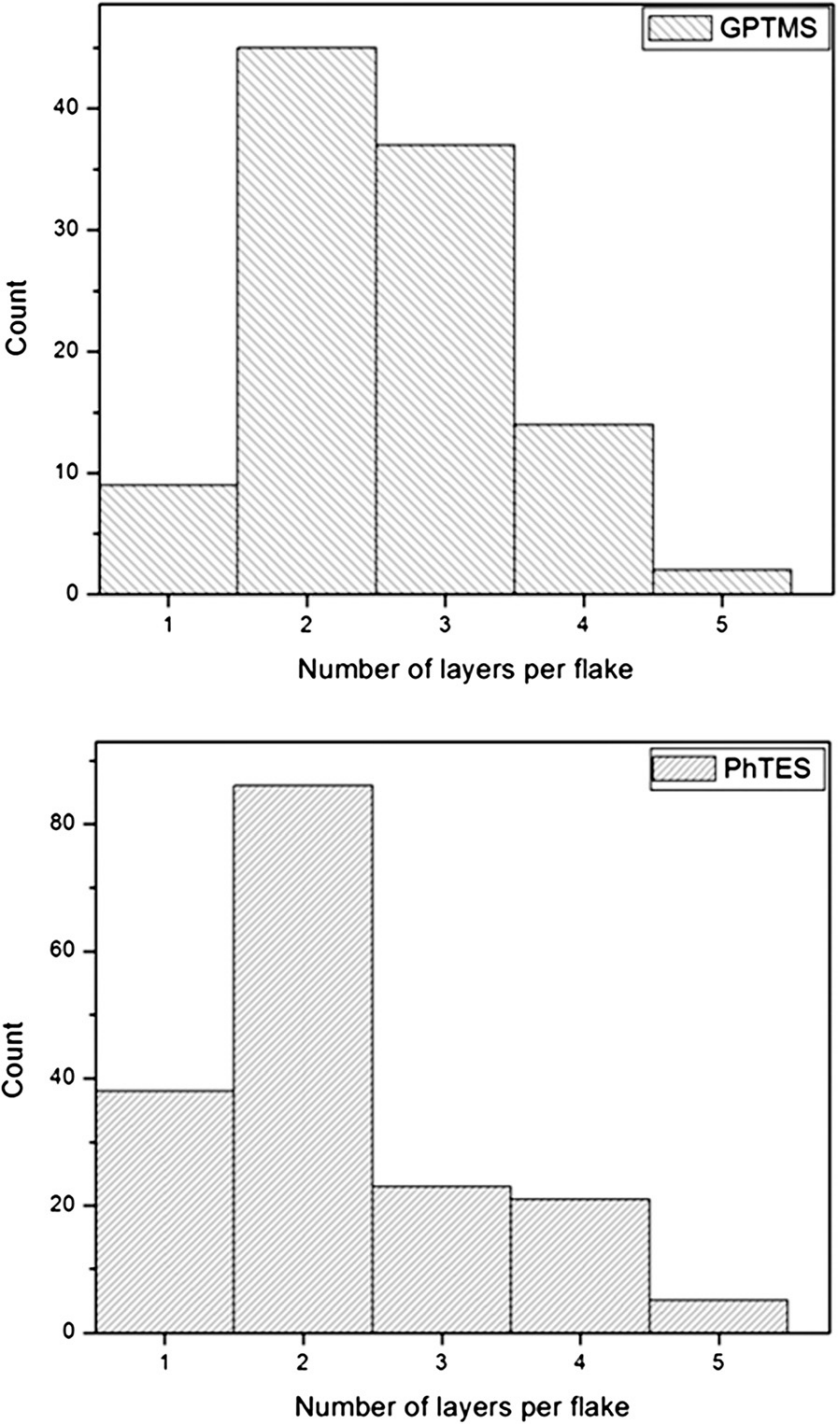
**Statistical analysis.** Histograms showing the number of layers per flake measured for graphene obtained by exfoliation of graphite in GPTMS (top) and PhTES (bottom).

A statistical analysis on TEM data was performed in order to verify the exfoliation, thus analyzing carefully the edge of the graphene flakes and measuring the number of layers presented in each flake [12]. At this regards about fifty different images have been observed in order to obtain a significant number of flakes for the statistical analysis. The results are reported in Figure 6.

The flakes present good exfoliation degree with an average number of layers of 2.7 for GPTMS and 2.4 for PhTES; standard deviation was about 0.4 and 0.5, respectively. In both cases, only 14% of the flakes were present more than three layers. Moreover, no more than five layers have been counted in very few flakes thus indicating a narrow dispersion. These results confirm that the exfoliation process was very effective.

**Figure 7:**
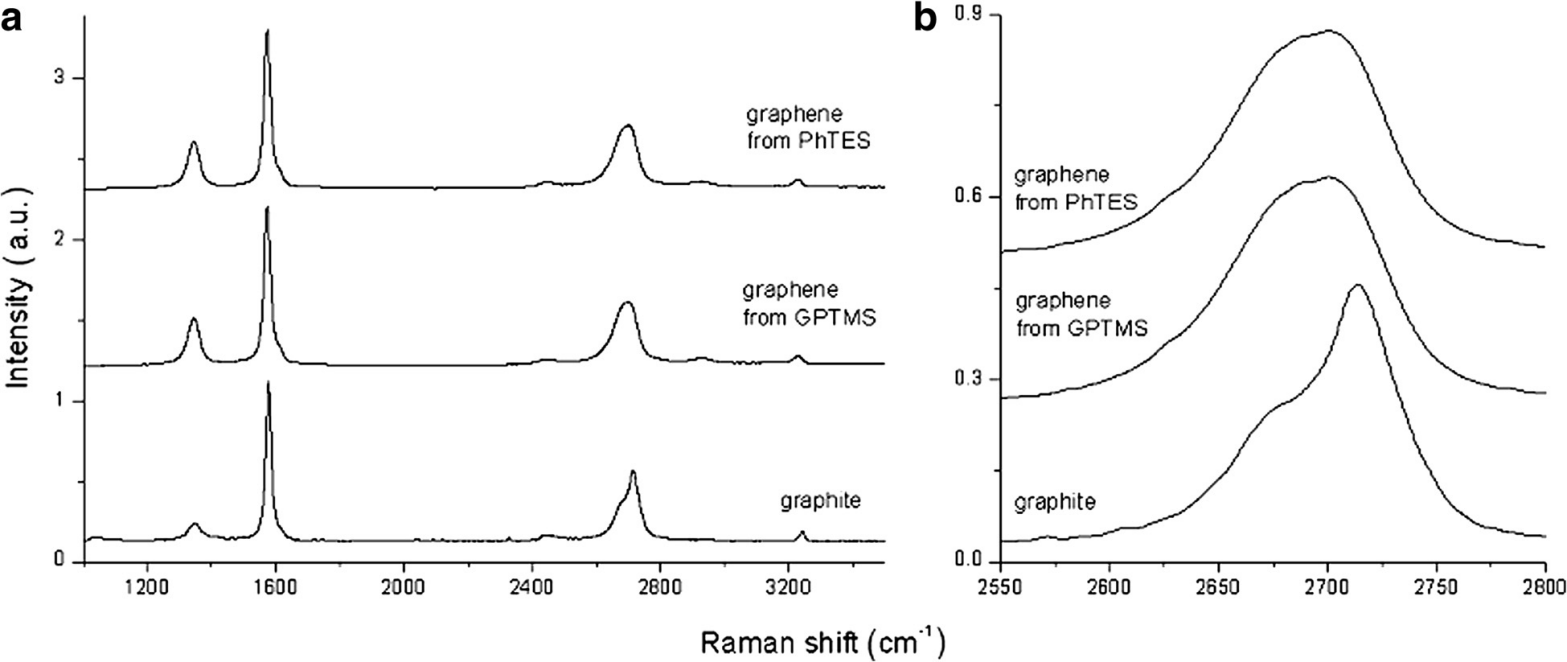
**Raman analysis.** Spectra of graphene obtained by sonication in PhTES and GPTMS from 5 wt.% of the initial graphite compared with graphite (**a**). 2D peaks evaluation for this systems (**b**).

Raman spectroscopy is essential for the characterization of graphene. Indeed, it is considered one of the best characterization techniques for discriminating between graphite and graphene [15,28-30]. As shown in Figure 7a, typical Raman signals of graphene recovered from the dispersions of GPTMS and PhTES are very similar, both exhibiting the characteristic graphene peaks. In particular, as far as PhTES is concerned, the G band at 1,577 cm^−1^, the 2D band at 2,696 cm^−1^, and the disorder-related D peak at approximately 1,346 cm^−1^ are evident. Similarly, graphene obtained from GPTMS shows the G band at 1,574 cm^−1^, the 2D band at 2,701 cm^−1^, and the disorder-related D peak at approximately 1,345 cm^−1^. The shape and position of the 2D peaks (Figure 7b) is typical of bilayer graphene (four components with the main peak at approximately 2,701 cm^−1^, as confirmed by a deconvolution process [30]). As a comparison, the 2D peak of graphite consists of two components and the main peak is upshifted to 2,713 cm^−1^.

The disorder-related D peak is present also in the initial graphite powder, but its intensity is higher for graphene. This finding was already reported in which graphene was produced by sonication of graphite and can be attributed to the new edges produced during the sonication process: Ultrasonic treatment causes the decrease in size of the flakes compared to the original graphite, with a consequent increase of the total edge length [12,24,31]. Comparing the intensity of the peaks and the *D*/*G* ratio found in the case of graphene obtained in GPTMS and PhTES, only little differences can be found, thus indicating that the disorder induced by the exfoliation process is very similar. Namely, the *D*/*G* ratio is 0.47 for graphene dispersed in GPTMS and 0.65 for that dispersed in PhTES, while the reference value for graphite powder is 0.14.

On the basis of the above concentration results, some considerations about the use of the Hildebrand solubility parameters *δ* should be done. Indeed, Hernandez et al. [13] stated that these parameters could be the key for envisaging the best graphene solvent media. In particular, they calculated a *δ* value for graphene equal to *ca*. 23 MPa^1/2^, this value being the same of *N*-methyl-2-pyrrolidone [12]. However, GPTMS, the best solvent medium reported here, is characterized by *δ* = 14.5 MPa^1/2^[32]. This value suggests that graphene solubility parameters should be revised and/or that they are not adequate for any reliable solubility prediction on this respect.

## Conclusions

A significant improvement of the methods used so far for graphene obtainment has been carried out. Namely, the two new liquid media in which graphene was found to effectively disperse were investigated. In particular, GPTMS and PhTES were chosen as representatives of the larger family of organosilanes. Both of them are commonly used in sol–gel synthesis; moreover, GPTMS can be also used for the preparation of polymer hybrids. In detail, this compound resulted to be one of the most effective medium found so far for dispersing graphene, allowing for a concentration of this material equal to 8.00 mg/ml.

The concentration value found for PhTES is much lower (0.66 mg/ml) but, if especially compared with that of most the reported data [11,22,26,27], it can be considered of interest; for instance, for the preparation of polymer composites, in which an even lower concentration of graphene might result in peculiar final properties of the resulting material [16,18,33-35].

## Competing interests

The authors declare that they have no competing interests.

## Authors’ contributions

DN prepared the first dispersions of graphene in organosilanes and also drafted the manuscript. RS and VA performed dispersion analysis. SS developed the dispersion method initially found by DN together with this latter. MP carried out the Raman analysis. LP and JMK performed TEM and statistical analysis. AM coordinated the work. All authors read and approved the final version of the manuscript.
